# Co-Delivery of Docosahexaenoic Acid and Brain-Derived Neurotropic Factor from Electrospun Aligned Core–Shell Fibrous Membranes in Treatment of Spinal Cord Injury

**DOI:** 10.3390/pharmaceutics14020321

**Published:** 2022-01-28

**Authors:** Zhuo-Hao Liu, Yin-Cheng Huang, Chang-Yi Kuo, Chi-Cheng Chuang, Ching-Chang Chen, Nan-Yu Chen, Ping K. Yip, Jyh-Ping Chen

**Affiliations:** 1 Department of Neurosurgery, Chang Gung Memorial Hospital, Linkou, Chang Gung University School of Medicine, Kwei-San, Taoyuan 33305, Taiwan; b8402022@gmail.com (Z.-H.L.); ns3068@gmail.com (Y.-C.H.); 8702047@cgmh.org.tw (C.-C.C.); ccc2915@cgmh.org.tw (C.-C.C.); 2 Department of Chemical and Materials and Materials Engineering, Chang Gung University, Kwei-San, Taoyuan 33302, Taiwan; onesky1997@gmail.com; 3 Department of Internal Medicine, Chang Gung Memorial Hospital, Linkou, Chang Gung University School of Medicine, Kwei-San, Taoyuan 33305, Taiwan; nellychen@gmail.com; 4 Centre for Neuroscience, Surgery & Trauma, Blizard Institute, Barts and The London School of Medicine and Dentistry, Queen Mary University of London, London E1 2AT, UK; p.yip@qmul.ac.uk; 5 Department of Plastic and Reconstructive Surgery and Craniofacial Research Center, Chang Gung Memorial Hospital, Linkou, Kwei-San, Taoyuan 33305, Taiwan; 6 Research Center for Food and Cosmetic Safety, College of Human Ecology, Chang Gung University of Science and Technology, Taoyuan 33302, Taiwan; 7 Department of Materials Engineering, Ming Chi University of Technology, Tai-Shan, New Taipei City 24301, Taiwan

**Keywords:** spinal cord injury, docosahexaenoic acid, brain-derived neurotrophic factor, electrospinning, regenerative medicine

## Abstract

To restore lost functions while repairing the neuronal structure after spinal cord injury (SCI), pharmacological interventions with multiple therapeutic agents will be a more effective modality given the complex pathophysiology of acute SCI. Toward this end, we prepared electrospun membranes containing aligned core–shell fibers with a polylactic acid (PLA) shell, and docosahexaenoic acid (DHA) or a brain-derived neurotropic factor (BDNF) in the core. The controlled release of both pro-regenerative agents is expected to provide combinatory treatment efficacy for effective neurogenesis, while aligned fiber topography is expected to guide directional neurite extension. The in vitro release study indicates that both DHA and BDNF could be released continuously from the electrospun membrane for up to 50 days, while aligned microfibers guide the neurite extension of primary cortical neurons along the fiber axis. Furthermore, the PLA/DHA/BDNF core–shell fibrous membrane (CSFM) provides a significantly higher neurite outgrowth length from the neuron cells than the PLA/DHA CSFM. This is supported by the upregulation of genes associated with neuroprotection and neuroplasticity from RT-PCR analysis. From an in vivo study by implanting a drug-loaded CSFM into the injury site of a rat suffering from SCI with a cervical hemisection, the co-delivery of DHA and BDNF from a PLA/DHA/BDNF CSFM could significantly improve neurological function recovery from behavioral assessment, as well as provide neuroprotection and promote neuroplasticity changes in recovered neuronal tissue from histological analysis.

## 1. Introduction

Traumatic spinal cord injury could lead to transient or permanent neurological dysfunction at or below the lesion site. Neurologic recovery from spinal cord injury (SCI) is hindered by a cascade of pathological events, including vascular disruption, ischemic change, inflammatory response, and ongoing apoptosis, following immediate mechanical trauma [1]. During the past decades, several approaches to spinal cord repair have been advocated to repair the neuronal structure and to enhance the neuronal functional recovery [2]. It is hypothesized that enhancing neuroprotection and synaptic plasticity would lead to patient neurologic recovery as a result of improved neural networks. For example, human gingival mesenchymal stem cells can provide an effective therapeutic approach toward motor neuron injury treatment and against scratch-injury-induced cell death [3], as well as treat SCI in a mouse model [4]. For pharmacological interventions of SCI, a drug can only target one aspect of the multifaceted pathophysiological change of SCI, usually making it difficult for clinical translation. Given the complex pathologic changes following SCI, the development of combinatory therapeutic approaches is, therefore, a trend for SCI treatment [5].

Solving an unmet clinical need is a primary motivation for biomedical engineering research. Electrospun membranes serve as an ideal scaffold for neuronal repair, due to their unique architecture, which is close to the structural organization of a natural extracellular matrix [6,7]. Specifically, delivering therapeutic agents by electrospun membranes can permit site-specific controlled drug release to overcome the difficulty of systemic delivery of therapeutics to a lesion site. Indeed, several studies have attempted the delivery of therapeutic agents through electrospun nanofibers for neuronal repair [8,9]. However, one factor that needs to be taken into consideration for neuronal repair is that the neuronal tract contains aligned and ordered bundles of axons, and such an oriented structure plays a vital role in the directional transmission of nerve conduct [10]. Therefore, to improve nerve recovery by enhancing neuronal extension and arrangement, current research focuses on the development of aligned fibers to guide neuronal extension [11,12,13].

Polyunsaturated fatty acids (PUFA) are lipids with major structural and signaling roles in the central nervous system (CNS). Especially docosahexaenoic acid (DHA), as the major omega-3 PUFA, is endowed with such functionality. Unsurprisingly, over the past decades, there has been expanding study of the health benefits of PUFAs, with evidence emerging that omega-3 PUFAs have huge therapeutic potential in various CNS disorders, including Zellweger syndrome, schizophrenia, depression, and Alzheimer’s disease [14]. The significant enhancement of the therapeutic effect of DHA on SCI treatment has also been reported in different animal models [15,16,17]. These studies demonstrated that DHA can provide a neuroprotective effect by ameliorating inflammation responses [18,19], decreasing oxidative stress [20,21], and reducing glutamate-induced cytotoxicity, both in vivo [22] and in vitro [23].

In addition to providing a neuroprotective effect, promoting neuroplasticity change in neuronal tissues plays a vital role in neurologic recovery following SCI. The brain-derived neurotrophic factor (BDNF) is an essential factor that plays a key regulatory role of plasticity in healthy and injured spinal cords. It was postulated that BDNF can ameliorate the damage of spinal cords and improve locomotive function through the sprouting of corticospinal tract fibers following SCI [24,25]. Other studies indicate that BDNF also has the ability to upregulate the expression of neuronal regeneration-associated genes, such as growth-associated protein 43 (GAP-43) and T-alpha-1-tubulin [26,27]. In vivo studies demonstrated that the local delivery of BDNF can lead to an increased number or diversity of fibers as well as synapse formations [28,29]

Previous studies have shown that DHA can increase BDNF synthesis and signaling in neuronal systems, which also influenced cannabinoid-mediated synaptic plasticity [30,31]. However, the efficacy of combinatory treatment with DHA and BDNF is not well delineated. To address this issue, we implanted a DHA/BDNF-loaded aligned core–shell fibrous membrane (CSFM) into the injury site in a cervical hemisection SCI animal model. Our results reveal that a DHA/BDNF dual-drug-loaded electrospun membrane can provide a neuroprotective effect, promote neuroplasticity changes, and enhance neurologic functional recovery.

## 2. Materials and Methods

### 2.1. Preparation of Core–Shell Fibrous Membranes (CSFM)

**Figure 1:**
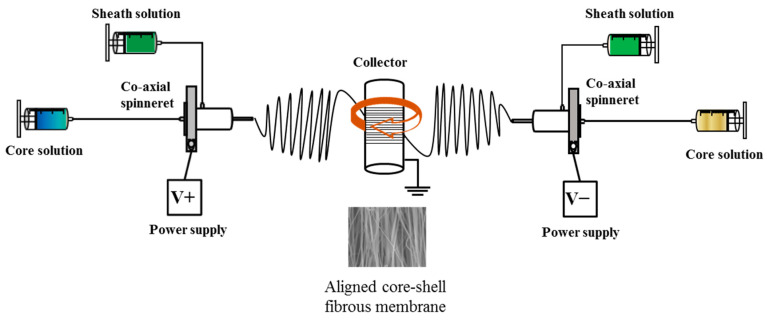
The schematic diagram showing the setup for preparing aligned core–shell fibrous membranes (CSFM) by electrospinning.

To fabricate drug-loaded CSFM, two co-axial spinnerets were used during the electrospinning process [32]. The shell spinning solution is 10% (*w*/*v*) poly(l-lactide-co-d,l-lactide) (PLA) (L-lactide:D,L-lactide = 70:30, intrinsic viscosity = 2.4 dL/g, Sigma–Aldrich, St. Louis, MO, USA), prepared in dichloromethane. The core spinning solution is distilled deionized water (ddH_2_O) for PLA fibers, 1 μM DHA (Sigma–Aldrich, St. Louis, MO, USA) in 99% ethanol for PLA/DHA fibers, and 250 ng/mL BDNF (Cloud-Clone Co., Katy, TX, USA) in ddH_2_O for PLA/BDNF fibers. The electrospun membrane was prepared by co-axial electrospinning by placing the spinnerets horizontally to either side of a rotating drum collector (Figure 1) [33]. One spinneret was used for preparing PLA/DHA electrospun fibers while the other for PLA/BDNF electrospun fibers. One spinneret was connected to a positive high voltage power supply while the other spinneret was connected to a negative high voltage power supply, both at 20–25 kV applied voltage. Using two syringe pumps to deliver the core and sheath solutions at 0.3 mL and 1 mL/h to each spinneret, electrospun fibers were collected on a grounded collector (covered with aluminum foil) rotating at 2600 rpm, and placed 15 cm from the needle tip in each spinneret. Three types of electrospun membranes were prepared: PLA CSFM with electrospun PLA fibers from each spinneret; PLA/DHA CSFM with electrospun PLA/DHA fibers from each spinneret; PLA/DHA/BDNF CSFM with electrospun PLA/DHA or PLA/BDNF fibers from each spinneret.

### 2.2. Characterization of Core–Shell Fibrous Membrane (CSFM)

The alignment and size of the fibers in the CSFM were determined by scanning electron microscopy (SEM) with a Hitachi S3000N scanning electron microscope (Tokyo, Japan). At least 100 fibers, randomly chosen from 10 images, were used to measure the fiber diameter and fiber angle distribution. To determine fiber alignment, the fiber orientation relative to a vertical direction (taken as 90°) was used to calculate the fiber angle distribution (from 0 to 180°). The core–shell structure of electrospun fibers was studied by transmission electron microscopy (TEM) with a JEM-1230 transmission electron microscope (Tokyo, Japan). To determine the release of DHA and BDNF from electrospun membranes, the PLA/DHA/BDNF CSFM were cut into small pieces (~10 mg) and incubated in 1 mL of pH 7.4 phosphate buffer solution (PBS) at 37 °C. The released solution was completely removed for analysis at predetermined time points, followed by adding 1 mL of fresh PBS to continue the drug release experiment up to 50 days. The amount of DHA and BDNF in PBS at each time point was determined separately by using enzyme-linked immunosorbent assay (ELISA) kits (Cloud-Clone Co., Katy, TX, USA), following the manufacturer’s protocols.

### 2.3. In Vitro Cell Culture

#### 2.3.1. Cytotoxicity

To examine the cytotoxicity of CSFM, an indirect contract method, following ISO 10993-5, the biological evaluation of medical devices, was used. In brief, sterilized, square-shaped CSFMs (1 × 1 cm^2^) were incubated in 1 mL of Dulbecco’s Modified Eagle Medium (DMEM) containing 10% (*v*/*v*) fetal bovine serum (FBS) for 24 h. The extraction medium was collected and used for the culture of 3T3 fibroblasts. The cells were seeded in a 24-well tissue culture plate (1 × 10^4^ cells per well) for 4 h and cultured with the extract for 24 h at 37 °C in a humidified 5% CO_2_ environment. The MTS assay (CellTiter 96^®^ AQueous One Solution Cell Proliferation Assay, Promega, Madison, WI, USA) was applied to measure the absorbance values at 492 nm, which were recorded using an ELISA plate reader and normalized to those of the control (cells cultured with culture medium) at each time point.

#### 2.3.2. Primary Cortical Cell Culture

The primary cortical neurons culture was performed from embryos of Sprague–Dawley rat brains (E18), adopting the methods described previously [34]. Cells were seeded on a polyethylenimine substrate in minimum essential medium containing Earle’s salts and supplemented with 10% heat-activated fetal bovine serum (FBS). After the cells settled down (3–4 h post-plating), the plating medium was replaced with modified Neurobasal medium. After washing and pelleting, cells were resuspended to 0.25 × 10^6^ cells/mL concentration and loaded onto a poly-D-lysine-coated glass coverslip, for incubation at 37 °C in 5% CO_2_. For the cell culture on the CSFM, a 100-μL cortical cell suspension (0.5 to 0.8 × 10^6^ cells/mL) was loaded to a CSFM. The cell-seeded membrane was incubated for 1–2 h before adding the culture medium. After culturing for 3 days, the cells were fixed and prepared for scanning electron microscopy and confocal microscopy examination.

#### 2.3.3. Reverse Transcription Polymerase Chain Reaction (RT-PCR)

To verify gene expression indicative of neurite extension, mRNA was extracted using Trizol and cDNA was generated from mRNA with a Total RNA Isolation Kit and Maxime RT PreMix Kit, according to standard protocols. The expression of neural marker genes, including growth-associated protein-43 (GAP-43), activating transcription factor 3 (ATF-3), and neurotrophin-3 (NT-3), were studied using glyceraldehyde 3-phosphate dehydrogenase (GAPDH) as a housekeeping gene.

#### 2.3.4. Neurite Outgrowth Evaluation

The length and number of neurite outgrowths from the primary cortical neurons were assayed to examine the effects of drug-loaded CSFMs. Isolated primary cortical cells were seeded onto glass cover slips coated with poly-d-lysine in a 24-well cell culture plate. After 1 day of cell culturing, we put a CSFM (1 × 1 cm^2^) at the bottom of an 8.0-μm Corning Transwell^®^ cell culture plate insert. A membrane-loaded insert was fitted into each well of the 24-well culture plate, followed by the addition of cell culture medium to fully immerse the membrane. The effects of the drug released from the PLA/DHA and PLA/DHA/BDNF CSFM during co-culture with cortical neurons were analyzed and compared with the PLA CSFM. Three days after in vitro cell culturing, we fixed the cells with 4% paraformaldehyde (20 min), permeabilized the cells with cold methanol, and thoroughly washed them with PBS. By incubating cortical neuronal culture with β-tubulin-III primary antibody (1:1000) for 2 h at room temperature for immunostaining, a secondary antibody, conjugated with AlexaFluor 488 (1:1000), was added for incubation at room temperature for 45 min. After PBS washes, cells were mounted with FluorSave reagent and observed under a confocal microscope. The length of neurite was determined from a line drawn along every neurite from each cell body, from which the average neurite length per neuron was calculated using ImageJ software. Control experiments were carried out by culture primary cortical cells in cell culture medium containing 3 μM DHA or 250 ng/mL BDNF.

### 2.4. In Vivo Study

#### 2.4.1. Animal Surgery

The female adult Sprague–Dawley rats (220–250 g) applied in this study were housed at the animal laboratory of Chang Gung Memorial Hospital under standard conditions. All procedures used during the animal experiments have been approved by the Ethics Committee (IACUC approval number CGU106-028) for the creation of the cervical spinal cord lateral hemisection animal model, following previously described methods [15,35]. The animals were deep-anesthetized via isoflurane and a longitudinal incision was performed in the midline to expose the cervical lamina at level C4-5. The spinal cord was identified after C4-5 laminectomy. A lateral hemisection was made with a microblade in the left of the cervical spinal cord. Three minutes after the spinal cord was lesioned, a CSFM was put over the lesion to cover the dura matter. Finally, the muscle and skin were sutured, and the rats were placed on a heating pad until full recovery before being taken back to their cage. Post-operative care involved a sub-cutaneous injection of analgesic and saline twice daily, for 3 days post-surgery. Six animals in each group were employed in this study to conduct behavioral assessment and histological analysis. The groups include: control group, receiving spinal cord hemisection only; PLA group, receiving spinal cord hemisection and with PLA CSFM implantation; PLA/DHA group, receiving spinal cord hemisection and with PLA/DHA CSFM implantation; DHA/BDNF group, receiving spinal cord hemisection and with PLA/DHA/BDNF CSFM implantation.

#### 2.4.2. Behavioral Assessment

The neurological function recovery was assessed using a staircase test and grid exploration tests. In order to evaluate the affected forelimb’s skilled function, the Montoya staircase test was applied. Briefly, the animals were trained to grasp the food pellets in the test apparatus 3 days per weeks for 2 weeks. The number of rats remained in the staircase boxes for 15 min, and the total numbers of food pellets eaten, displaced, or remaining on the steps on each side were recorded. The test was performed every 2 days after surgery for 7 days. After 7 days, the test was performed every other day for 21 days. The grid exploration test was used to assess spontaneous skilled locomotion and limb movement involved in precise stepping, coordination, and paw placement of animals. Before the surgery, the animals were placed on the grid twice prior to surgery for habituation and baseline scores. Rats were placed on a wired rung and allowed to freely explore for 5 min. The test was videotaped and an observer blinded to the treatment group scored the number of foot-slips out of the first 30 in forelimbs, and 20 in the hindlimbs. Rats were tested on day 7, 14, and 21 post-surgery.

#### 2.4.3. Histological Analysis

Following euthanasia 3 weeks postoperatively, the spinal cord tissues were harvested from all experimental groups following perfusion with saline solution and paraformaldehyde solution. The spinal cords were post-fixed in paraformaldehyde overnight and subject to cryopreservation with 30% sucrose before freezing. Spinal cord sections were cut horizontally at a thickness of 15 μm. For immunohistochemistry analysis, frozen spinal cord sections were randomly chosen for immunostaining. The sections were washed in PBS for 5 min each three times at room temperature, and incubated in blocking solution for 30 min. Sections were labelled overnight, with neuronal nuclei antigen (NeuN), serotonin (5-HT), or synaptophysin primary antibody, separately. The slides were then washed three times with PBS and incubated with AlexaFluor 488 or 594 conjugated secondary antibody for 2 h at room temperature. After washing again with PBS three times, sections were cover-slipped and images were obtained from fluorescent microscopy. The ImageJ software was used for quantification, which was carried out by observers blinded to the experiment design.

### 2.5. Statistical Analysis

For behavioral data and immunostaining, a two-way repeated-measures ANOVA was applied to compare the effects. For quantitative RT-PCR analysis of gene expression, a one-way analysis was used to compare the treatment effect among various groups.

## 3. Results

### 3.1. Preparation and Characterization of Electrospun Aligned Core–Shell Fibrous Membranes (CSFM)

**Figure 2:**
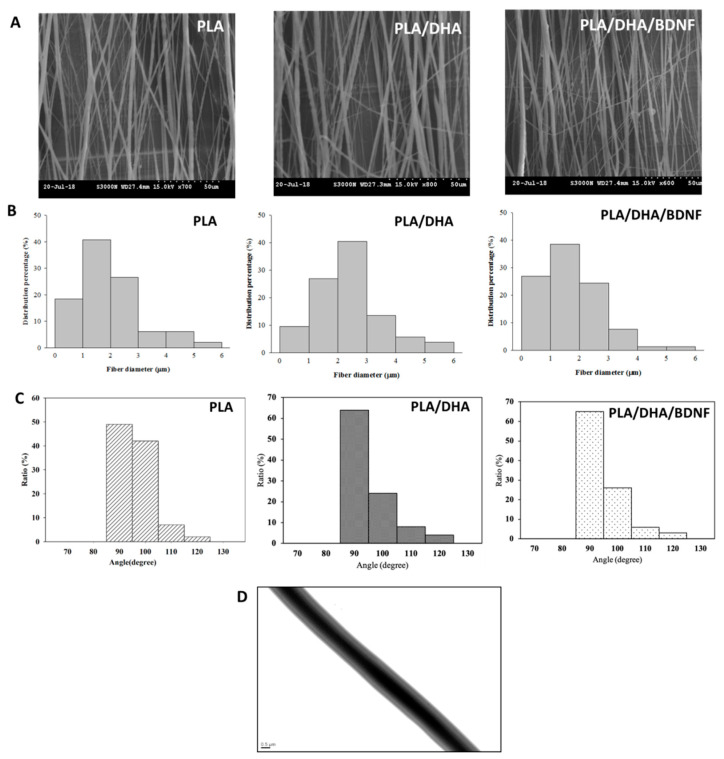
The scanning electron microscopy images (**A**), bar = 50 μm, the fiber diameter distribution (**B**), and the fiber angle distribution (**C**) of PLA, PLA/DHA, and PLA/DHA/BDNF core–shell fibrous membranes (CSFMs). The transmission electron microscopy (TEM) (**D**) bar = 0.5 μm, image of PLA/DHA/BDNF CSFM.

The SEM images of the CSFM shows that microfibers were well arranged and formed a dense array (Figure 2A). The fiber diameter distribution is shown in Figure 2B, from which the average fiber sizes were calculated to be 2.01 ± 1.09, 2.43 ± 1.14, and 1.80 ± 0.96 μm for the PLA, PLA/DHA, and PLA/DHA/BDNF membranes, respectively, with no significant difference found among them. While some misalignment was still observed in the fiber assemblies, most of the fibers (>90%) were oriented within 10° of the vertical direction (90°) (Figure 2C). The TEM image of electrospun fibers further confirms the core–shell structure (Figure 2D).

**Figure 3:**
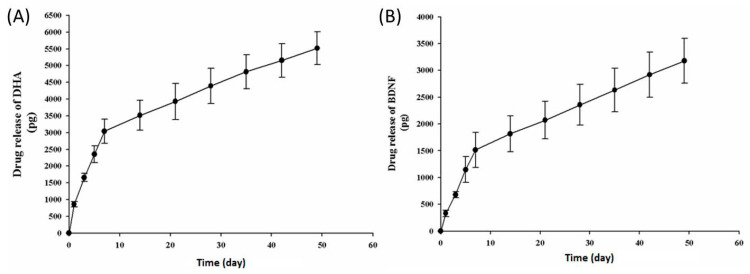
The cumulative weight of DHA (**A**), and BDNF (**B**) released from PLA/DHA/BDNF core–shell fibrous membranes (CSFMs).

The release behaviour of encapsulated DHA and BDNF plays a vital role in the practical application the electrospun membranes for SCI treatment. Therefore, the in vitro release profile of DHA and BDNF from PLA/DHA/BDNF CSFM was studied by measuring the cumulative released weight of each drug in PBS at 37 °C. The concentration of DHA and BDNF in PBS was determined using ELISA for the accurate detection of each component individually. As shown in Figure 3, two-stage drug release behaviour was observed for both DHA and BDNF, where an initial burst release in 7 days was followed by sustained drug release for up to 50 days. The drug release profile appears to follow the zero-order kinetics with a linear relationship between the cumulative drug concentrations vs. time. The drug concentration will remain constant in the solution during both stages, albeit the first stage will provide a higher drug concentration during the cell culture. Using the flow rates and concentrations of DHA, BDNF, and PLA solutions during the preparation of the membranes by electrospinning, we can calculate the quantity of DHA and BDNF in a 10-mg membrane sample, which was used in the drug release experiments, to be 9.9 ng and 7.5 ng, respectively. With the calculated drug loading in the membrane, the cumulative release percentages of DHA and BDNF, at the end of the drug release experiments, were calculated to be 53.3% and 42.7%, respectively. Therefore, the drug was still releasing after 50 days and the cumulative drug release weight did not reach a plateau at 100%, as shown in Figure 3.

### 3.2. In Vitro Studies

**Figure 4:**
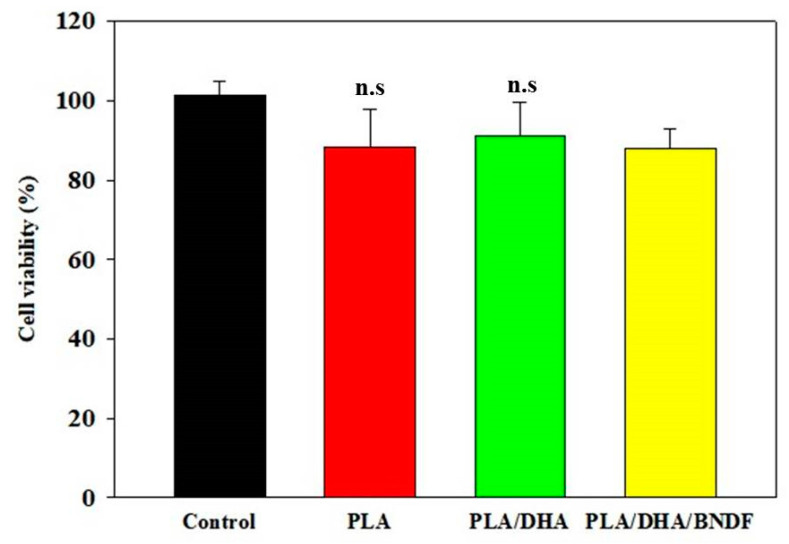
The cytotoxicity of PLA, PLA/DHA, and PLA/DHA/BDNF core–shell fibrous membranes (CSFMs). The fibroblasts were cultured with 24-h extract of a CSFM. The cell viability was compared with fibroblasts cultured with fresh cell culture medium (control), which was taken as 100%. The MTS assay was used to determinate the relative cell viability after cell culture for 24 h. n.s., not significant compared with control (*p* > 0.05).

Evaluation of the possible cytotoxicity of the CSFM was carried out according to the ISO 10993-5 standard, using the 24-h extract of a membrane for cell culture. After culturing fibroblasts with the extract for 24 h, the cell viability was determined from MTS assays, where cells cultured with fresh cell culture medium were taken as a control. As shown in Figure 4, a higher-than-90% cell viability was found for all CSFMs, indicating the CSFMs are cyto-compatible. Furthermore, components in both the core and shell compartments, as well as the toxic organic solvents used for electrospinning, will not induce noticeable cytotoxicity, as PLA, DHA, and BDNF are non-toxic to cells and dichloromethane in the shell layer was removed during the electrospinning process.

**Figure 5:**
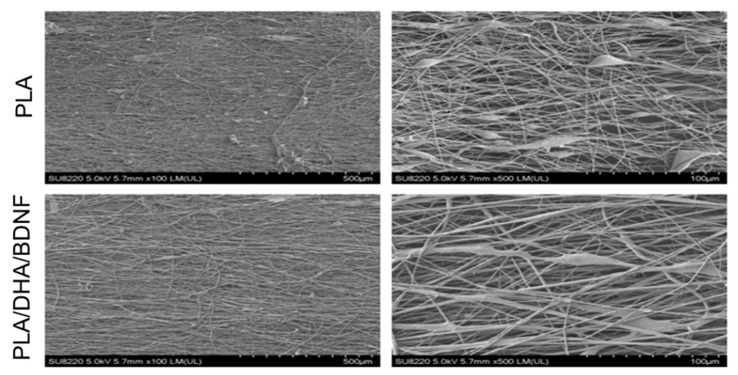
The scanning electron microscopy images of primary neurons cultured on aligned drug-free PLA core–shell fibrous membranes (CSFMs) and drug-loaded PLA/DHA/BDNF CSFMs.

To determinate the efficiency of an aligned CSFM in guiding neuronal outgrowth, primary cortical neuronal cells was cultured on drug-free and drug-loaded CSFMs. The cell morphology and neurite extension were investigated by SEM. As shown in Figure 5, neuronal cells are well-attached to aligned fibers, showing an elongated shape parallel to the fiber orientation. The observed directional neurite growth can be attributed to the directional topography of the fibers, guiding cell growth along the fiber axial direction.

**Figure 6:**
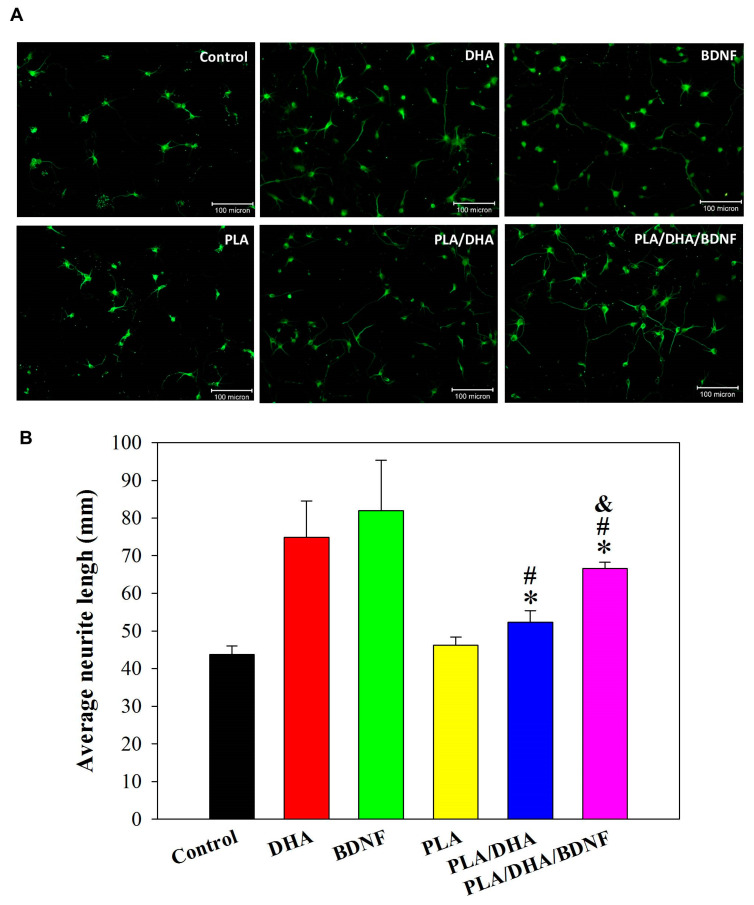
(**A**) The observation of neurite outgrowth from β-tubulin-III-positive primary cortical neuron from fluorescent microscopy by culturing primary cortical neurons in cell culture medium containing 3 μM DHA or 250 ng/mL BDNF, and co-culturing primary cortical neurons with aligned core–shell fibrous membranes (CSFMs) in culture medium for 3 days (bar = 100 μm). (**B**) The average neurite length for each neuron from neurite outgrowth. * *p* < 0.05 compared with control; ^#^ *p* < 0.05 compared with PLA; ^&^ *p* < 0.05 compared with PLA/DHA.

In an attempt to test whether drug-loaded CSFMs can directly affect elongation of existing neurites, neurons were stained with an antibody against β-tubulin-III, a specific marker for the somatodendritic compartment. The β-tubulin III-positive cells from fluorescence staining were categorized into neurons, and their neurite extension was determined from the microscopy image. A positive control, by culturing primary cortical neuron in cell culture medium containing 3 μM DHA or 250 ng/mL BDNF, validates that each free drug can induce longer neurite extensions than the control (cell culture medium) (Figure 6). For neurons co-cultured with a CSFM, neurons co-cultured with the DHA/BDNF-loaded CSFM (PLA/DHA/BDNF) show the greatest neurite extension in comparison with other groups (Figure 6). The neurite extension length is significantly longer for PLA/DHA/BDNF than for drug-free PLA and DHA-loaded PLA/DHA CSFMs, with PLA CSNF showing insignificant differences from the control, as expected.

**Figure 7:**
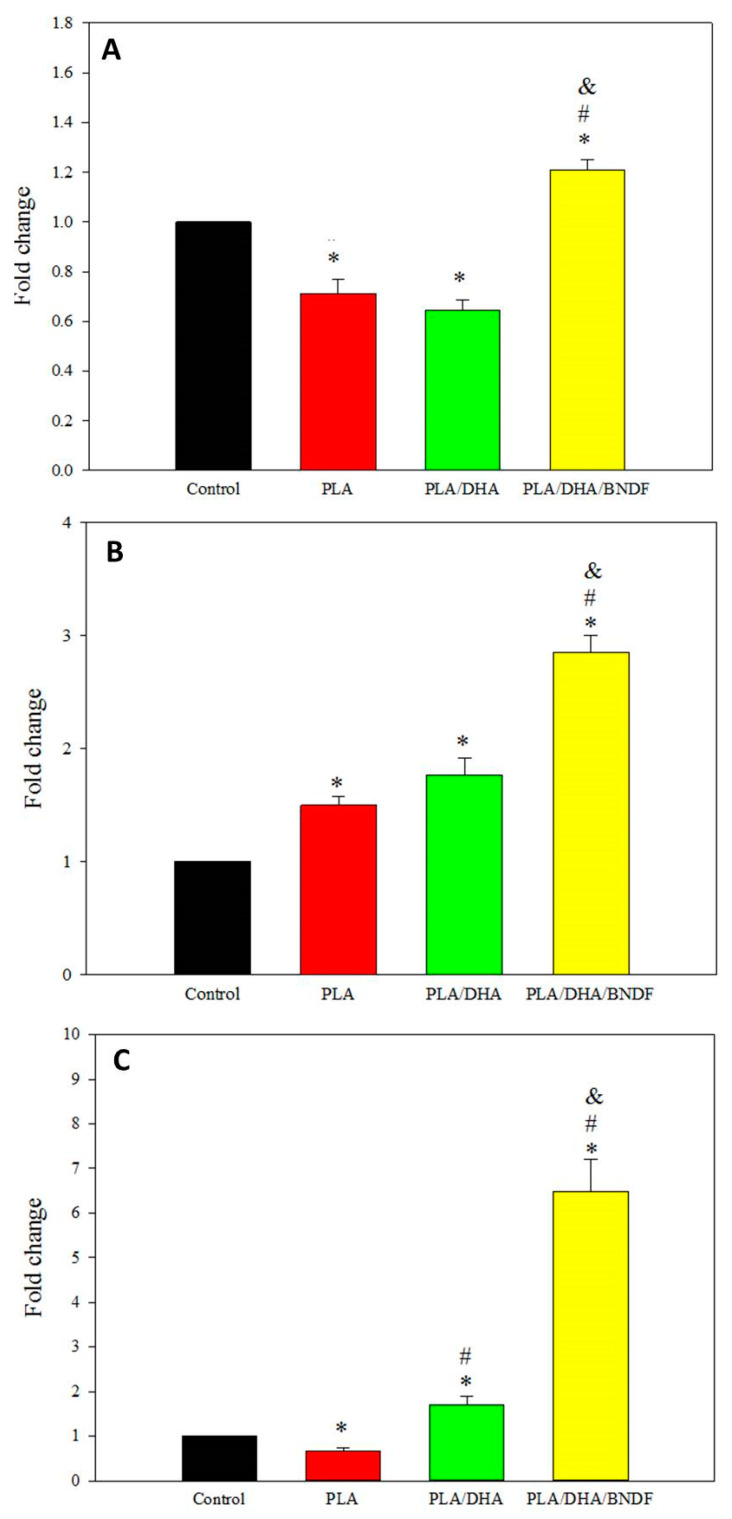
The relative mRNA expression of growth-associated protein-43 (GAP-43) (**A**), activating transcription factor-3 (ATF-3) (**B**), and neurotrophin-3 (NT-3) (**C**). The expression of these genes associated with neurite outgrowth was determined from real-time polymerase chain reaction (RT-PCR) by co-culturing primary cortical neurons with PLA, PLA/DHA, and PLA/DHA/BDNF core–shell fibrous membranes (CSFMs). The control is primary cortical neurons cultured with cell culture medium. * *p* < 0.05 compared with control; ^#^ *p* < 0.05 compared with PLA, ^&^ *p* < 0.05 compared with PLA/DHA.

To evaluate the possible mechanism to promote neurite growth after treatment with a drug-loaded CSFM, the expression of genes associated with neurite outgrowth was quantified after co-culturing primary cortical neurons with a CSFM for 3 days. The results shown in Figure 7 indicate that DHA can lead to significantly higher NT-3 gene expression for the PLA/DHA group over the control and PLA groups. Nonetheless, only the PLA/DHA/BDNF group demonstrates significantly upregulated gene expression for all genes associated with neurite growth, where a significantly higher level of GAP43, ATF-3, and NT3 gene expression was found from RT-PCR analysis (Figure 7).

### 3.3. In Vivo Studies

**Figure 8:**
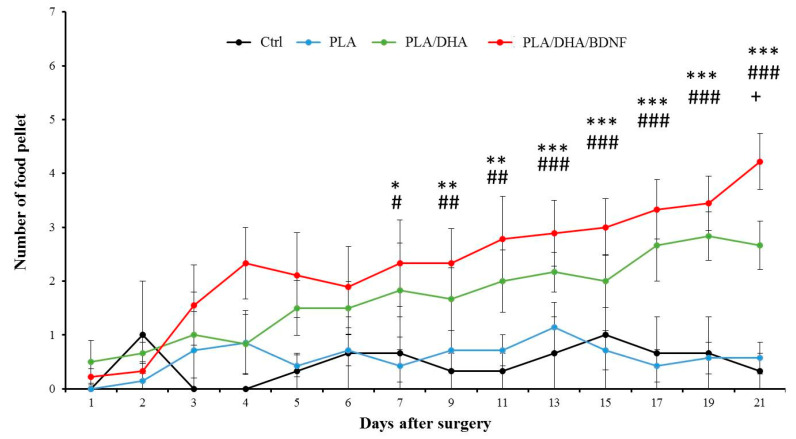
The evaluation of neurological function recovery by the Motaya staircase test. * *p* < 0.05, ** *p* < 0.01, *** *p* < 0.001 PLA/DHA/BDNF vs. control; ^#^ *p* < 0.05, ^##^ *p* < 0.01, ^###^ *p* < 0.001 PLA/DHA/BDNF vs. PLA; ^+^ *p* < 0.05 PLA/DHA/BDNF vs. PLA/DHA. The Ctrl is control group.

To access the neurologic functional loss and recovery in SCI animals receiving CSFM implantation, the Motaya staircase test and the grid exploration test were applied to evaluate the functions of four limbs following hemisection cervical SCI. The Motaya staircase behavioral test provides a sensitive measurement of skilled reaching, which was used here to assess reaching performance in rat cervical SCI model (Figure 8). All animals learned to collect food pellets from the staircases during the training period (two weeks). After cervical hemisection, a significant reduction of the number of eaten pellets was noted on day 1 among all four groups. Overall, the rats receiving drug-loaded CSFM treatment showed gradual improvement compared with the control and PLA groups. The SCI rats implanted with PLA/DHA/BDNF CSFM showed significant improvement in food-retrieval on day 7 over the control and PLA groups. Furthermore, no improved performance was observed throughout the behavioral test time in the control and PLA groups, with no significant difference found between them. Compared with the PLA/DHA group, the PLA/DHA/BDNF group showed consistent improvement in gross forelimb function from the staircase test over time, although no significant improvement in food pellet collection ability was noted until day 19. However, the rats in the PLA/DHA/BDNF group showed significantly improved performance compared with the other three groups at 3 weeks after cervical hemisection. Taken together, our results indicate that combining the DHA/BDNF treatment with PLA/DHA/BDNF electrospun membranes enabled neurologic recovery in comparison with other treatments.

**Figure 9:**
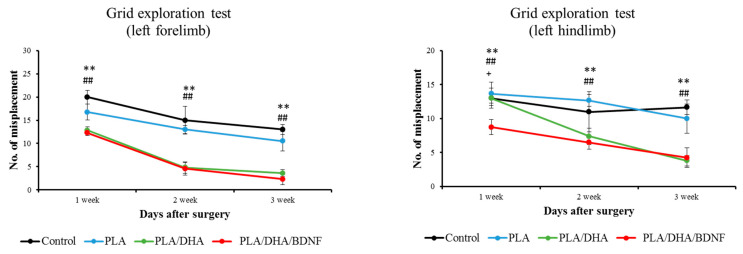
The evaluation of the neurological function recovery by the grid exploration test. ** *p* < 0.01 PLA/DHA/BDNF vs. control; ^##^ *p* < 0.01 PLA/DHA/BDNF vs. PLA; ^+^ *p* < 0.05 PLA/DHA/BDNF vs. PLA/DHA.

As we know, the cervical injury can result in neurologic deficits in forelimb and hindlimb functions. To examine the recovery of neurologic functions with a different behavioral test, we used the grid exploration test to determine fine control of the forelimb and hindlimb locomotion of rats with SCI. As shown in Figure 9, 3 weeks following SCI injury, the rats receiving treatment with either PLA/DHA or PLA/DHA/BDNF CSFMs made significantly fewer foot-slips in the forelimb, compared with the control and PLA groups. In regard to hindlimb locomotion, the rats receiving PLA/DHA/BDNF implantation can substantially reduce the hindlimb misplacement one week post-surgery. At 3 weeks post-surgery, the performance in the grid exploration test demonstrates that both PLA/DHA and PLA/DHA/BDNF can lead to significant improvement over the PLA and control groups, although no significant difference was found between PLA/DHA and PLA/DHA/BDNF (Figure 9).

**Figure 10:**
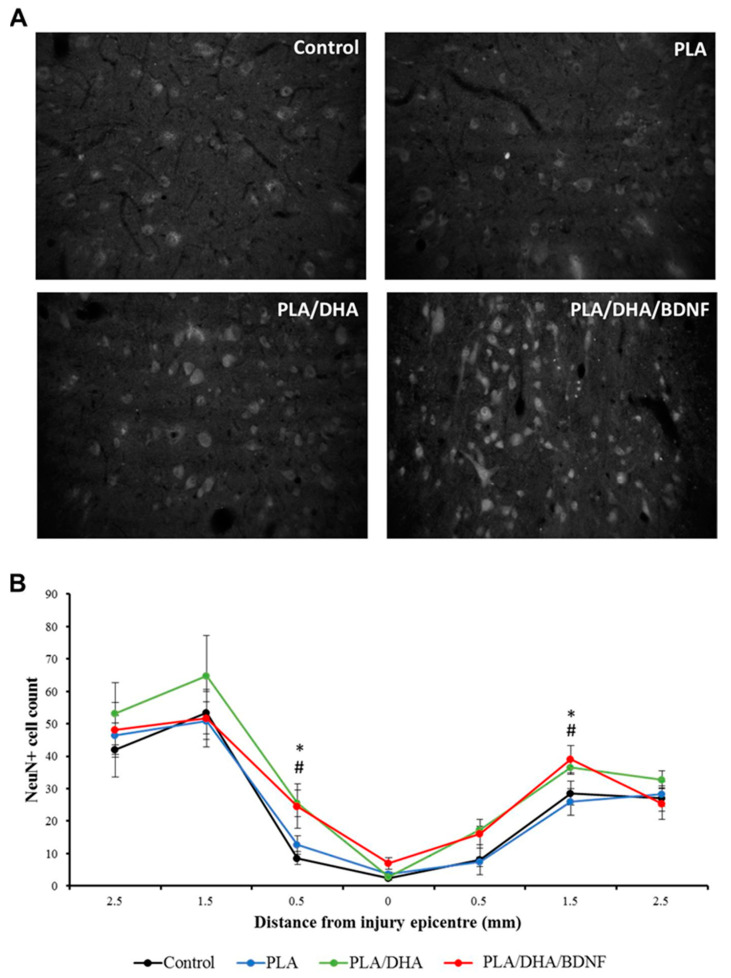
The immunolabeling of neuronal nuclei antigen (NeuN) from the spinal cords of SCI rats after different treatments (**A**), and the calculated number of NeuN-positive cells in the region rostral and caudal to the injury site (**B**). * *p* < 0.05 PLA/DHA/BDNF vs. control; ^#^ *p* < 0.05 PLA/DHA/BDNF vs. PLA.

To determine whether CSFM can enhance neuron survival via a neuroprotective effect within the cervical spinal cord, we quantified the sparing neuronal cells by immunostaining a neuronal biomarker, neuronal nuclei antigen (NeuN), in the region rostral and caudal to the injury site. After cervical hemisection, a loss of neurons was identified in the epicenter region in each group (Figure 10A). The quantification analysis revealed significantly higher neuronal survival in the region rostral and caudal to the epicenter area in the PLA/DHA and PLA/DHA/BDNF groups at the end of the experiment (Figure 10B). Furthermore, higher neuronal survival in the region 1.5-mm rostral to the lesion was also observed in the PLA/DHA/BDNF group when compared with the other three groups. Our results demonstrate that DHA/BDNF co-loaded CSFMs may provide the best neuroprotective effect against neuronal loss after SCI.

**Figure 11:**
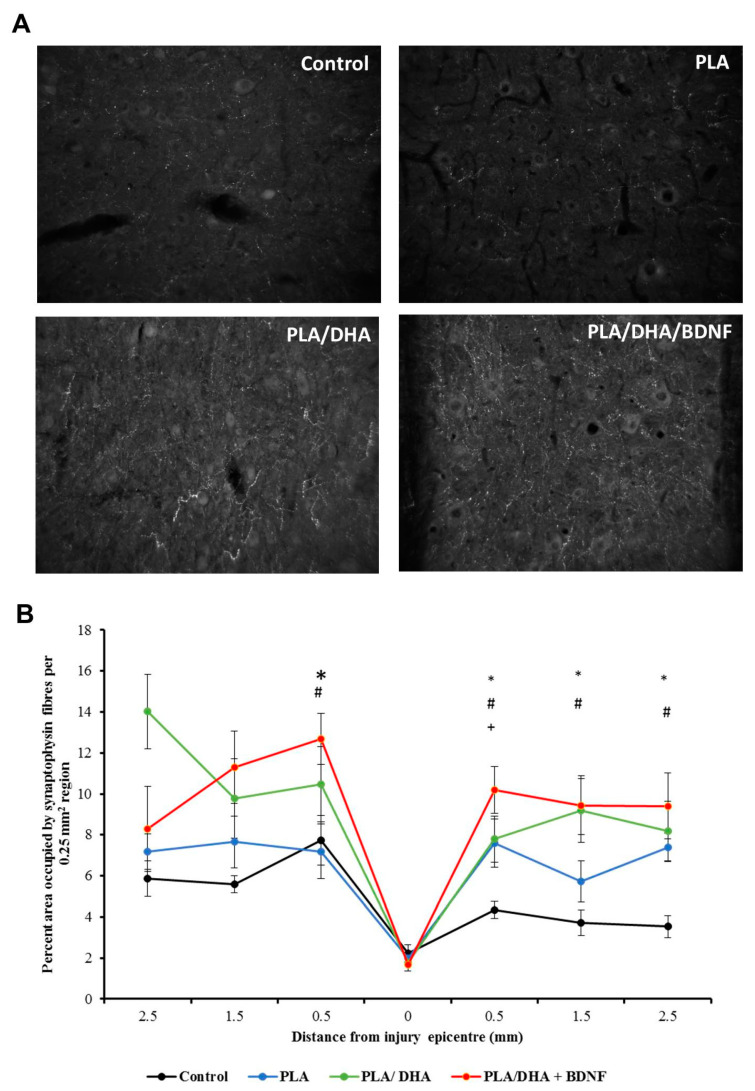
The immunolabeling of serotonin (5-HT) from the spinal cords of SCI rats after different treatments (**A**), and the area percentage occupied by the 5-HT fiber in the region rostral and caudal to the injury site (**B**). * *p* < 0.05 PLA/DHA/BDNF vs. control; ^#^ *p* < 0.05 PLA/DHA/BDNF vs. PLA; ^+^ *p* < 0.05 PLA/DHA/BDNF vs. PLA/DHA.

Serotonin (5-HT) is a neuromodulator secreted by supraspinal neurons that initiate spinal locomotor pathways. Upregulation of serotonin fiber distribution contributes to the improvement of motor function after treatment. At 3 weeks post-injury, longitudinal sections show a marked depletion of serotonin fibers in the epicenter region and caudal to the lesion site (Figure 11A). Serotonin fibers in the rostral part of the lesion site were significantly increased in both drug-loaded CSFM groups, where no significant difference was found between the control and PLA groups (Figure 11B). Furthermore, significantly increased serotonin in the region 0.5-mm caudal to the lesion site was noted for the PLA/DHA/BDNF group, where quantification of 5-HT axons revealed significantly increased serotoninergic fibers.

**Figure 12:**
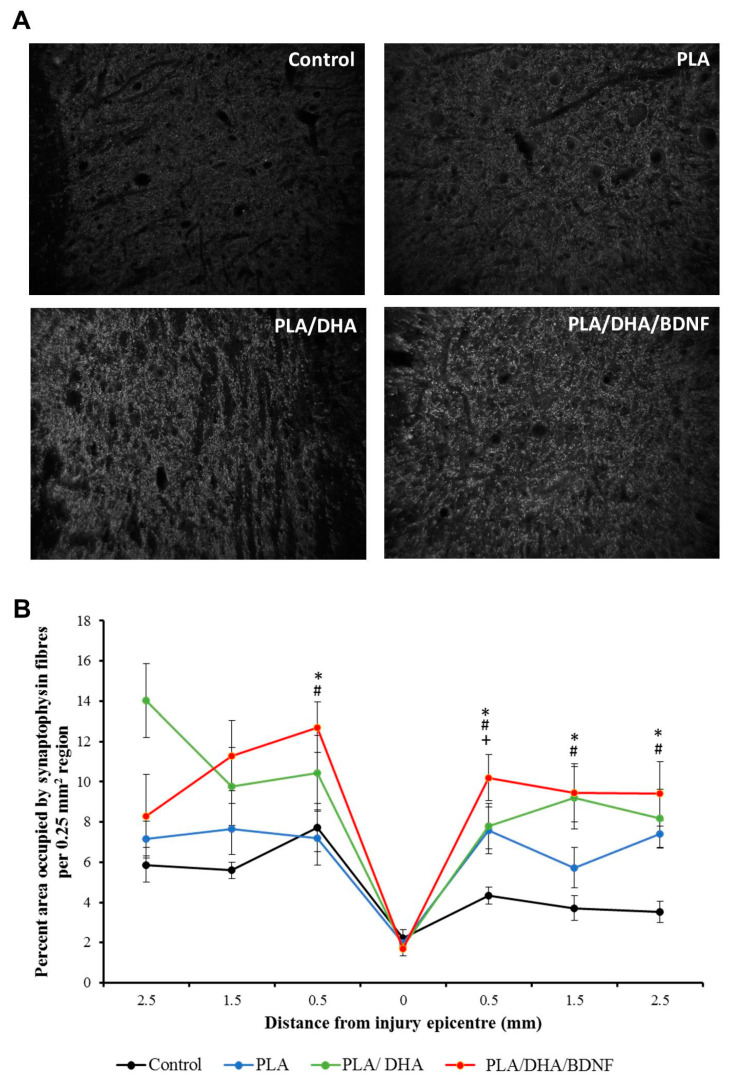
The immunolabeling of synaptophysin from the spinal cords of SCI rats after different treatments (**A**), and the area percentage occupied by the synaptophysin fibers in the region rostral and caudal to the injury site (**B**). * *p* < 0.05 represents PLA/DHA/BDNF vs. control; ^#^ *p* < 0.05 PLA/DHA/BDNF vs. PLA; ^+^ *p* < 0.05 PLA/DHA/BDNF vs. PLA/DHA.

Synaptophysin is the synaptic vesicle protein to evaluate the synaptic function after injury. To evaluate the ability of CSFM to strengthen the synaptic function after injury, we quantified the expression of synaptophysin in the grey matter of a spinal cord. As shown in Figure 12A, the analysis of synaptophysin immunoreactivity revealed significantly more synaptic terminals in PLA/DHA and PLA/DHA/BDNF groups. In the region 0.5-mm caudal to lesion site, the PLA/DHA/BDNF treatment significantly enhanced the synaptophysin fiber expression (Figure 12B).

## 4. Discussion

### 4.1. The Use of Aligned CSFM in SCI Treatment

Spinal cord injury is a catastrophic event that may lead to permanent disability. However, therapeutic treatment of SCI is limited due to multi-faceted pathological events during various phases, including primary and secondary injury. The treatment approach may differ in different stages of this disease. Therefore, therapeutic strategies for SCI tend to use multifunctional systems that combine various treatment methods to overcome this limitation and enhance neurologic recovery.

In our study, an aligned CSFM was designed as a feasible membrane-type vehicle for the delivery of DHA and BDNF in SCI treatment, which could be easily applied in a clinical setting. The delivery of bioactive agents and/or drugs to the injured spinal cord is classically achieved by using invasive intrathecal pumps and microinjection devices, which may lead to scarring formation and spinal cord compression [36]. Drug-release from electrospun membranes is a well-established drug-delivery method to allow for a controlled drug-release rate over an extended period of time, but it has rarely been explored for SCI treatment. The CSFM offers several advantages in drug delivery, such as the controlled release of incorporated agents, versatility in drug selection, and biodegradability. Clinically, the drug-loaded CSFM could be implanted in the spinal cord during decompression/fixation surgery of SCI patients so that no secondary CSFM implant surgery is required for drug administration. The application of this biomaterial also allows us to provide sustainable delivery of a drug and a neurotrophic factor at the same time to achieve enormous therapeutic effect. In this regard, aligned CSFMs provide an alternative approach to repeated drug injections for the sustained release of two therapeutic agents simultaneously, and expedite a less-invasive drug-delivery practice.

The implantation of biodegradable materials may offer a novel alternative approach for the continuous bolus injection of active drugs or neurotrophic factors. However, randomly arranged electrospun fibers might hinder the neuronal cell axon growth. Recently, there has been a surge of research attempting to characterize the role of aligned electrospun membranes for neuronal regeneration. Therefore, we used aligned CSFMs to enhance neurite linear arrangement in this study. From the SEM image, a PLA/DHA/BDNF CSFM can promote the neurite outgrowth of seeded primary neuronal cells, which might enhance neurologic recovery following SCI. Although the electrospun membranes contain aligned microfibers rather than nanofibers, this fiber size is actually favorable for extending neurite length. It is reported that aligned electrospun fibers with higher fiber diameters (>750 nm) can preferentially direct the neurite extension of primary neurons to result in a longer neurite length along the fiber axis than using aligned fibers with smaller diameters (<750 nm) [37].

Considering the drug-release study, the solubility of DHA in PBS is 0.1 mg/mL and the solubility of BDNF in PBS is >1 mg/mL. The concentration of DHA and BDNF in the release medium (PBS) is in the order of ng/mL (Figure 3). Therefore, the release medium is not saturated with DHA or BDNF, even when choosing 1 mL as the volume of the dissolution medium. For the accurate detection of DHA and BDNF concentrations by ELISA, the choice of this volume can also provide absorbance values within the detection limit while fulfilling the sink condition. Our dual-drug-delivery system can consistently release active DHA and BDNF from the cores of electrospun fibers, which can promote neurite extension, as observed from SEM and confocal microscopy images. Furthermore, in SCI animal models, our data show that the synergic effect from encapsulated DHA and BDNF can enhance neurite extension, provide neuroprotective effects, and promote neuroplasticity changes, compared to DHA treatment alone.

### 4.2. DHA Improves SCI Recovery

Current pharmaceutical strategies for the treatment of SCI focus on the prevention of secondary injury progression by reducing inflammation, oxidation stress, and apoptosis [38]. Recently, there has been a substantial body of evidence indicating that omega-3 PUFAs are neuroprotective. Highly polyunsaturated fatty acids are abundant in the membrane phospholipids in the nervous system and play an essential role in the maintenance of nervous function. The DHA is a long-chain omega-3 PUFA that contributes 50% of PUFA in the CNS membrane. DHA is also the mediator involved in the resolution of inflammation and provides endogenous neuroprotection. There is substantial agreement that DHA can provide neuroprotective effects in SCI through anti-inflammatory response, anti-oxidation effect, and signaling transduction [21]. In a preclinical study, DHA was demonstrated to improve neurologic function in a SCI animal model, either by acute intravenous injection or via dietary supplement [35,39,40,41]. However, the slow onset of DHA treatment or oral dietary without initial acute bolus did not confer neuroprotection effects after SCI [21,40]. It is postulated that an insufficient amount of DHA may reach the spinal cord tissue via the oral route during the acute phase. Therefore, rapidly increasing the DHA level in the spinal cord tissue appears to be essential during an early critical time window, which may be one possible scenario to overcome such a limitation. Nonetheless, one study revealed that the neuroprotective efficacy of the acutely administrated DHA can be synergistically enhanced through oral DHA supplement for 6 weeks following SCI [21]. Hence, maintaining an appropriate level of DHA in the spinal cord tissue seems to be necessary for significant neuroprotection in SCI. From the release profiles, the DHA was released during an initial burst, where about half of the encapsulated DHA was liberated within 10 days. This was followed by the sustained release of DHA for up to 50 days. Hence, our aligned CSFM can meet our therapeutic requirement of DHA in SCI treatment with well-controlled drug release.

### 4.3. Synergic Effects of DHA and BDNF on Neurologic Recovery in SCI

The BDNF is a member of the neurotrophic factor and a potent modulator of neural plasticity. The BDNF-mediated axonal growth, reflected from the increased number or diversity of fibers extending into a tissue graft, has been frequently observed [42]. A previous study has shown that BNDF acts through the TrKB pathway and plays a vital role in neuroplasticity following SCI, including neuronal regeneration or sprouting [43]. The exogenous delivery of BDNF in SCI models enhances axonal growth and neuron survival by intrathecal infusion [44], intraspinal viral transduction [45,46], and fetal spinal tissue [47]. Furthermore, the sustained release of BDNF from biomaterials has provided evidence to promote neurologic function in SCI [48] and stroke [49] animal models.

However, the efficacy of combining DHA and BDNF treatment in SCI has not been well explored. Expanding evidence is pointing out that DHA can upregulate the BDNF expression via free fatty acid receptors [50,51]. The BDNF/TrkB signaling pathway is one of the different neurobiological mechanisms of action which have been proposed to promote the neurologic function recovery in SCI. Recent evidence also postulated the involvement of n-3 PUFAs with the BDNF/tyrosine kinase receptor B (TrkB) signaling pathway [52,53]. Previously, we developed a DHA-encapsulated electrospun membrane to provide a neurotrophic microenvironment for enhancing neurologic function recovery [54]. In the current study, we extended the results from the previous study to reveal the efficacy of SCI treatment by the co-delivery of DHA and BDNF with an aligned CSFM. Our results can shed light on the synergistic effect of the fiber topography and sustained release of DHA/BDNF for enhancing spinal cord regeneration ability through quantitative analysis in animal models.

Following SCI, various changes of gene expression are related to neurologic function recovery. In order to verify the gene expression related to neuroprotection and neuroplasticity, the expression levels of ATF-3, GAP-43, and NT-3 genes were examined. The ATF-3 is a member of activating transcription factor/cAMP-responsive element binding proteins (ATF/CREN). It has been reported that ATF-3 upregulation could be found during the development of rubrospinal neurons regenerating axons into peripheral nerve grafts in the cervical spinal cord [55]. Therefore, the overexpression of ATF-3 is frequently connected to neuroprotection, which acts as a transcriptional repressor. The GAP-43 is another crucial protein involved in neurite outgrowth. Lipids such as DHA appear to support growth in cultured hippocampal neurons and regulate expression or proregenerative genes such as GAP-43 [56,57]. The GAP-43 gene is also known as an important mediator of the neurotrophic effects of BDNF on neuronal survival and plasticity [58,59]. Using combination BDNF/DHA therapy, the drug-loaded CSFM is shown to provide significant neurologic recovery by upregulating these genes. The NT-3 has also been reported to exhibit growth-stimulating and chemo-attractive roles in neurological recovery by enhancing axonal growth and synaptic function in neuronal circuits [60,61]. In the group treated with DHA/BDNF-loaded CSFM, the expression of NT-3 can be highly upregulated, which might contribute to neuroplasticity effect in SCI rats.

## 5. Conclusions

Our study proposes the practicability of delivering two different agents simultaneously using electrospun fibrous membranes for enhanced neurologic improvement in SCI. The sustained release of DHA and BDNF was demonstrated to be extended for up to 50 days. Compared to single-compound treatment in vitro, the co-delivery of DHA and BDNF can achieve prominent neurite outgrowth through upregulating neuron-regeneration genes. Aligned fibrous mats also contribute towards the axonal growth in seeded neuron cells. In a SCI animal model, CSFM-mediated drug delivery resulted in the capacity for providing neuroprotection and promoting neuroplasticity changes in neuronal tissue, which can enhance the neurologic improvement. Collectively, our designed dual-agent delivery system can be utilized to evaluate the therapeutic effects of various drug combinations in different clinical settings.

## Data Availability

The data presented in this study are available on request from the corresponding author.
